# Factors influencing on-scene time in a rural Norwegian helicopter emergency medical service: a retrospective observational study

**DOI:** 10.1186/s13049-017-0442-5

**Published:** 2017-09-21

**Authors:** Øyvind Østerås, Jon-Kenneth Heltne, Bjørn-Christian Vikenes, Jörg Assmus, Guttorm Brattebø

**Affiliations:** 1 Department of Anaesthesia and Intensive Care, Haukeland University Hospital, PO Box 1400, 5021 Bergen, Norway; 2 Department of Clinical Medicine, Faculty of Medicine, University of Bergen, PO Box 7804, 5020 Bergen, Norway; 3 Centre for Clinical Research, Haukeland University Hospital, PO Box 1400, 5021 Bergen, Norway; 4 Norwegian National Advisory Unit on Trauma, Division of Emergencies and Critical Care, Oslo University Hospital, PO Box 4950 Nydalen, 0424 Oslo, Norway

**Keywords:** On-scene time, Scene time, Helicopter, Hems, Air ambulances, Emergency medical services, First hour quintet, Norway

## Abstract

**Background:**

Critically ill patients need to be immediately identified, properly managed, and rapidly transported to definitive care. Extensive prehospital times may increase mortality in selected patient groups. The on-scene time is a part of the prehospital interval that can be decreased, as transport times are determined mostly by the distance to the hospital. Identifying factors that affect on-scene time can improve training, protocols, and decision making. Our objectives were to assess on-scene time in the Helicopter Emergency Medical Service (HEMS) in our region and selected factors that may affect it in specific and severe conditions.

**Methods:**

This retrospective cohort study evaluated on-scene time and factors that may affect it for 9757 emergency primary missions by the three HEMSs in western Norway between 2009 and 2013, using graphics and descriptive statistics.

**Results:**

The overall median on-scene time was 10 minutes (IQR 5–16). The median on-scene time in patients with penetrating torso injuries was 5 minutes (IQR 3–10), whereas in cardiac arrest patients it was 20 minutes (IQR 13–28). Based on multivariate linear regression analysis, the severity of the patient’s condition, advanced interventions performed, mode of transport, and trauma missions increased the on-scene time. Endotracheal intubation increased the OST by almost 10 minutes. Treatment prior to HEMS arrival reduced the on-scene time in patients suffering from acute myocardial infarction.

**Discussion:**

We found a short OST in preselected conditions compared to other studies. For the various patient subgroups, the strength of association between factors and OST varied. The time spent on-scene and its influencing factors were dependent on the patient’s condition. Our results provide a basis for efforts to improve decision making and reduce OST for selected patient groups.

**Conclusions:**

The most important factors associated with increased on-scene time were the severity of the patient’s condition, the need for intubation or intravenous analgesic, helicopter transport, and trauma missions.

**Electronic supplementary material:**

The online version of this article (10.1186/s13049-017-0442-5) contains supplementary material, which is available to authorized users.

## Background

Patients suffering from a severe illness or injury require immediate prehospital assessment, appropriate treatment, and, in many cases, rapid transport to the hospital accompanied by competent personnel. A European project accentuated the so-called “First Hour Quintet” (cardiac arrest, respiratory failure, trauma, acute coronary syndrome, and stroke) as critical conditions of great importance in prehospital emergency care [1]. Many studies have assessed on-scene time (OST), but not all have found an association with mortality. Prolonged OST seems to increase mortality for trauma patients, however not in all settings and conditions [2]. The value of shortening the prehospital time has not received similar attention in medical emergencies, but reducing the interval between diagnosis and treatment for stroke and myocardial infarction seems beneficial [3, 4].

The backbone of Norwegian prehospital emergency medical care is ground ambulances and on-call general practitioners in the municipalities. An important supplement is the physician-staffed emergency medical services, including the helicopter emergency medical service (HEMS) [5]. An on-scene HEMS physician does not necessarily increase the OST, though more advanced interventions may be initiated [6–8]. The OST is the prehospital time interval that can be reduced, as transport times are mostly determined by the distance to the hospital. The main factors affecting OST have been described for trauma patients, but not specifically for all five conditions in the First Hour Quintet [9–13]. Clarifying these factors may improve decision making and treatment protocols, and provide a basis for targeted training, aiming to reduce OST in specific missions.

Our objectives were to assess OST in the HEMS and to investigate whether selected factors affect it in four specific and severe conditions in which a short OST was anticipated. Cardiac arrest patients were also assessed for comparison, with an increased OST anticipated for this group.

## Methods

### Study design and setting

This is a retrospective cohort study designed to investigate OST in the three HEMS bases in Førde, Bergen, and Stavanger, which cover the western region of Norway. The catchment area of these services is rural and includes islands, fjords, mountains, rough terrain, and narrow roads, as well as two major cities, Stavanger and Bergen. The total population is approximately 1.1 million on 45,000 km^2^ (17,400 mi^2^) of land, an area equivalent to mainland Denmark [14, 15]. Outside the cities, the population density is 15 persons/km^2^.

The Norwegian HEMS operates day and night year-round and may choose to respond with a rapid response car rather than a helicopter when the scene is nearby or the weather conditions prohibit the use of a helicopter. A ground ambulance is most often present on-scene when the HEMS arrives and offers an alternative mode of transportation to the hospital [16]. The helicopters (Eurocopter, EC 135 P2) are staffed with a pilot, a rescue paramedic, and a specially trained anesthesiologist and have capacity for one supine and one sitting patient. The HEMS in western Norway has been described previously in more detail [16, 17].

### Data source and management

On missions, the individual physician documented data in a paper-based form, which was subsequently registered in a database called “Airdoc” (Filemaker 8, Filemaker Inc., CA, USA). Landing and take-off times were also available immediately after each mission from data recorded by the pilot. All primary emergency HEMS missions, using a helicopter or rapid response car, with patient encounters from 2009 through 2013 were included in the analysis. Search and Rescue (SAR) missions and inter-hospital transfers were excluded. Patients who were entrapped when the HEMS arrived were also excluded from the analysis if transport was delayed due to the entrapment (Additional file 1). A free-text field in the mission report was assessed in all such cases.

### Methods and measurements

Our primary outcome was the OST and associated factors in five patient subgroups. We analyzed variables available in our database or through additional questions to the HEMS physicians. An overview of the variables included and the reason for exclusion is available from the corresponding author. OST was defined as the time from the patient encounter to the start of patient transport from the scene (i.e., when the patient’s stretcher started moving). Information about the mission, prehospital times, and patient data (vital signs, treatments performed, patient condition, and a free-text field) were available in the database. Advanced interventions were defined as interventions that could not be performed by the ground ambulance crew (e.g., intubation/tracheostomy; mechanical ventilation; thoracostomy/chest drain; chest compression device; thoracic needle decompression; external cardiac pacing; anesthesia; central venous, arterial, or intraosseous cannulation; use of neonatal incubator; nerve blocks; ultrasound; blood products; and the use of drugs not administered by the ground ambulance crew alone). All HEMS physicians involved in missions during the study period reported the year they became a specialist in anesthesiology. Darkness was defined for each mission according to civil twilight for the dispatch time, date, and latitude/longitude for the scene (center of the municipality). Unexpected, extreme, or missing values were assessed by reading the free-text fields and by cross-checking other data sources, such as the Emergency Medical Call Centre (EMCC) records and pilots’ flight logs. Values that were clearly incorrect were replaced if reliable data could be determined; otherwise, the values were excluded. When Glasgow Coma Scale (GCS) data were missing, a normal value (GCS = 15) was used in the analysis.

Five patient subgroups were selected for further analysis: acute myocardial infarction, stroke, head injuries, penetrating torso injuries, and cardiac arrest. In 1980, the National Advisory Committee for Aeronautics (NACA) score (Additional file 2) was modified for use in severity scoring of prehospital medical emergencies and trauma, and it is currently used by Norwegian HEMS [18]. To ensure that the selected patients were in fact severely ill or injured, cases with NACA 0–3 (none or no serious conditions) and NACA 7 (dead on-scene or during transport) were excluded. We hypothesized that the observed OST would be longer for cardiac arrest and shorter for the other groups, compared to overall OST.

### Analysis

We used descriptive methods to characterize the sample and OST for the subgroups and graphics (histograms) to illustrate distributions. The effects of factors were assessed for each of the subgroups by graphical methods and linear regression models using the OST as the outcome variable. The models were built in three steps, separately for each group. First, we estimated the unadjusted model for each factor. Next, we estimated the fully adjusted model containing all factors. In the third step, we estimated the final model containing all factors with a *p*-value <0.1 in one of the previous steps, in addition to age and gender. For all subgroups except penetrating torso injuries, we used a linear mixed effects model adjusted for HEMS base and with a random intercept for the individual doctor. The size of the penetrating torso injuries subgroup was too small to do the same, so we estimated a simple linear model. The significance level was set to 0.05. Descriptive statistics were calculated using IBM SPSS Statistics for Windows, Version 22 (IBM Corp., Armonk, NY), and the linear model using R 3.3 package nlme [19, 20]. The graphics were created using Matlab 7.10 (The MathWorks Inc., Natick MA, USA).

## Results

**Fig. 1:**
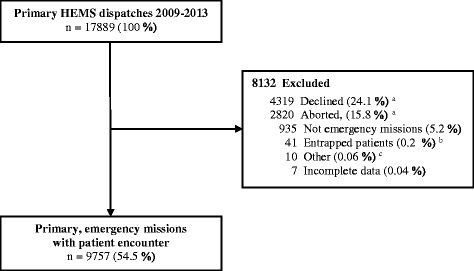
Flow chart showing all primary HEMS dispatches, with excluded and completed missions. Primary missions were defined as responses to patients outside hospitals. ^a^Declined dispatches or aborted missions were due to medical indication no longer being present, weather, concurrent missions, unable to perform a flight, or other reasons; 109 of the declined and 33 of the aborted missions (total 0.8% of the dispatches) were transferred to another HEMS in the area. Therefore, these incidents are reported as two separate dispatches. ^b^The characteristics of the 41 entrapped patients are presented in Additional File 1. ^c^HEMS base very close to the incident, completed without using a vehicle

**Fig. 2:**
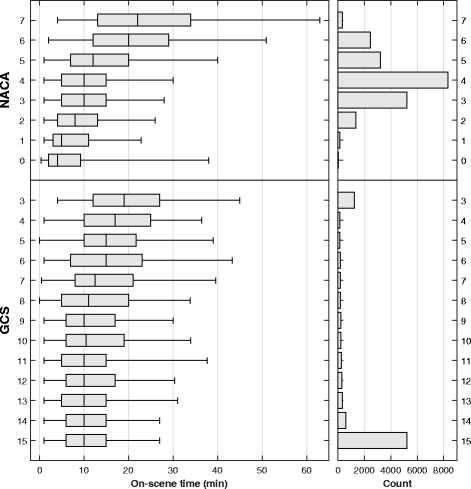
On-scene times and distribution of GCS and NACA in primary emergency missions (*N* = 9757). The boxes illustrate median, quartile 25 and quartile 75 on-scene times for the various values of Glasgow Coma Scale (GCS) values and National Advisory Committee for Aeronautics (NACA) scores. Whiskers indicate 5-, and 95-percentile. Missing GCS values (*n* = 5436) were replaced with a normal value (GCS = 15)

A total of 9757 emergency primary missions with patient encounters occurred during the study period (Fig. 1). The overall median OST was 10 min (IQR 5–16). Table 1 shows the patient characteristics. Higher NACA scores and lower GCS values were associated with an increase in OST (Fig. 2).

**Table 1:** Patient characteristics and on-scene time in primary emergency missions with patient encounter (N = 9757)

	All	NACA score = 0–3	NACA score = 4–6	NACA score = 7	Subgroups with NACA score of 4–6				
					Acute myocardial infarction	Stroke	Head injury^a^	Penetrating torso injuries	Cardiac arrest^a^
N (proportion of all)	9757 (100%)	3022 (31.0%)	5448 (55.8%)	1115 (11.4%)	777 (8.0%)	458 (4.7%)	348 (3.6%)	59 (0.6%)	683 (7.0%)
Median OST, minutes (IQR)	10 (5–16)	10 (5–16)	10 (5–14)	20 (13–31)^b^	9 (5–13)	8 (5–14)	11 (6–17)	5 (3–10)	20 (13–28)
Median age, years (IQR)	51.0 (27.0–67.0)	35 (16–57)	55 (32–69)	64 (48–75)	64 (54–73)	69.5 (57–79)	36 (20–58)	31 (24–39)	64 (52–73)
<18 years, N (%)	1495 (15.7%)	771 (26.7%)	665 (12.4%)	143 (3.3%)	1 (0.1%)	0	68 (19.8%)	5 (8.9%)	15 (2.2%)
Male gender, N (%)	6412 (66.2%)	1738 (58.8%)	3726 (68.4%)	810 (72.6%)	617 (79.4%)	267 (58.3%)	257 (73.9%)	46 (78.0%)	494 (72.3%)
Trauma, N (%)	2950 (30.1%)	1364 (45.1%)	1343 (24.7%)	159 (14.3%)	0	0	348 (100%)	59 (100%)	0
Advanced interventions, N (%)	4058 (41.6%)	595 (19.7%)	2725 (50.0%)	682 (61.2%)	557 (71.7%)	98 (21.4%)	134 (38.5%)	21 (35.6%)	603 (88.3%)
Intubated by HEMS, N (%)	1353 (13.9%)	8 (0.3%)	850 (15.6%)	479 (43.0%)	10 (1.3%)	54 (11.8%)	89 (25.3%)	5 (8.5%)	409 (59.9%)
Median GCS (IQR)	14 (6–15)	15 (15–15)	14 (6–15)	3 (3–3)	15 (15–15)	13 (9–15)	11 (6–14)	15 (14–15)	3 (3–3)

Missing data are presented in Additional file 8. ^a^On-scene time (OST) was significantly different from each of the other subgroups (*p* < 0.001; Kruskall-Wallis and Dunn’s Multiple Comparison Test). ^b^For 982 patients with a NACA score of 7, OST was missing, as the start of patient transport from the scene was not relevant for patients who were pronounced dead on scene. Patients suffering cardiac arrest were in most cases transported after ROSC were achieved. In a few cases, transported was initiated with continuous CPR using a chest compression device

**Fig. 3:**
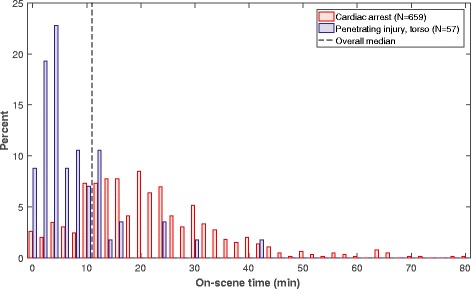
Distribution of on-scene time in cardiac arrest (*N* = 659) and penetrating torso injuries (*N* = 57). The overall median refers to the median OST in the five subgroups, 11 min. Patients suffering cardiac arrest were in most cases transported after ROSC were achieved. In a few cases, transported was initiated with continuous CPR using a chest compression device

OST varied between the five selected patient subgroups and was significantly different in both head injuries and cardiac arrest subgroups compared to all other groups (Table 1). The largest difference in OST was found between the penetrating torso injuries and cardiac arrest subgroups. The different distributions of OST are shown in Fig. 3.

**Fig. 4:**
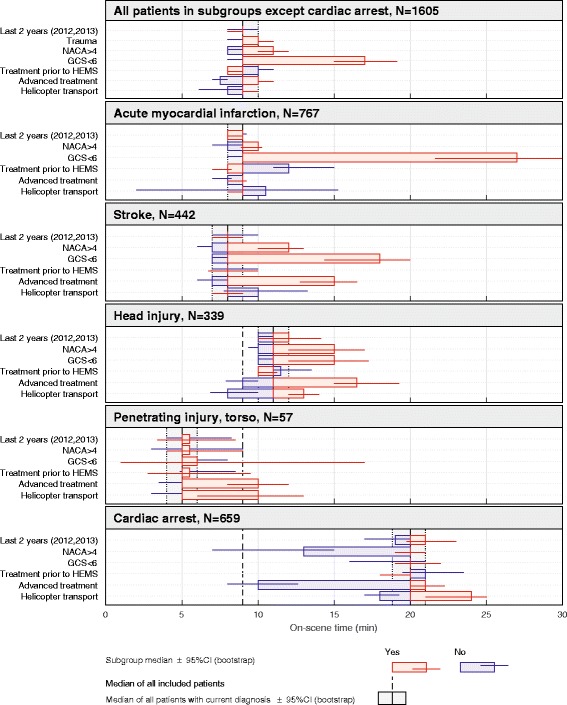
On-scene time and affecting factors (dichotomous) in subgroups of primary emergency missions with patient encounter (*N* = 2372). The subgroups included patients with a NACA score of 4–6 only. “Median of all included patients” refers to the median OST, 9 min, in all patients in subgroups except cardiac arrest (top panel). In the cardiac arrest subgroup, 647 (94.7%) patients were classified by a NACA score of 6. Patients suffering cardiac arrest were in most cases transported after ROSC were achieved. In these patients, a low NACA or a high GCS indicates successfully resuscitation before HEMS arrived, as our GCS and NACA variable describes the patient’s condition during HEMS patient care. In a few cases, transported was initiated with continuous CPR using a chest compression device

Figure 4 displays factors affecting OST in the five subgroups using dichotomous variables. Advanced treatment and a more severe condition based on the GCS and NACA score were associated with increased OST. For patients suffering from cardiac arrest, no advanced treatment and a low NACA score were associated with reduced OST. The factor helicopter transport was associated with increased OST in the head injuries, penetrating torso injuries, and cardiac arrest subgroups, whereas treatment prior to HEMS arrival was associated with reduced OST in the subgroup with acute myocardial infarction.

Multivariate linear regression analysis identified age, NACA score, helicopter transport, the use of intravenous analgesics, treatment prior to HEMS arrival, intubation, year in study period, and trauma missions as factors associated with significantly altered OST when including four patient groups (i.e., excluding cardiac arrest; Table 2). Gender, GCS, physician’s experience, and daylight were not identified as factors affecting OST. Median transport time in these cases was 25 min (IQR 16–35). The multivariate linear regression analyses for each subgroup are presented in Additional files 3, 4, 5, 6 and 7.

**Table 2:** Factors affecting on-scene time (linear mixed effect model)

Predictor	Unadjusted model *N* = 1605			Fully adjusted model *N* = 1597			Final model^b^ *N* = 1599		
	B	95% CI	p-value	B	95% CI	p-value	B	95% CI	p-value
Year in study period^a^	−0.31	(−0.60, −0.01)	0.040	−0.43	(−0.72, −0.14)	0.004	−0.42	(−0.67, −0.16)	0.001
Daylight (yes)	0.00	(−0.84, 0.83)	0.992	0.34	(−0.39, 1.06)	0.367	–	–	–
Age	−0.01	(−0.03, 0.01)	0.310	0.03	(0.01, 0.05)	0.008	0.03	(0.01, 0.05)	0.005
Male gender (yes)	−0.16	(−1.04, 0.71)	0.713	−0.26	(−1.02, 0.50)	0.505	−0.26	(−1.02, 0.50)	0.504
Trauma (yes)	1.96	(1.04, 2.87)	<0.001	1.61	(0.59, 2.63)	0.002	1.60	(0.58, 2.62)	0.002
NACA score	3.88	(3.25, 4.51)	<0.001	1.44	(0.77, 2.11)	<0.001	1.44	(0.78, 2.11)	<0.001
Glasgow Coma Scale	−0.70	(−0.82, −0.58)	<0.001	0.02	(−0.14, 0.17)	0.838	0.02	(−0.14, 0.17)	0.846
Treatment prior to HEMS (yes)	−1.12	(−2.00, −0.23)	0.014	−1.68	(−2.46, −0.90)	<0.001	−1.68	(−2.46, −0.90)	<0.001
Experience (years as specialist)	−0.06	(−0.19, 0.08)	0.409	0.01	(−0.14, 0.15)	0.921	–	–	–
Analgesics (yes)	5.68	(4.86, 6.49)	<0.001	3.06	(2.24, 3.87)	<0.001	3.07	(2.25, 3.88)	<0.001
Intubation (yes)	12.41	(11.22, 13.60)	<0.001	9.75	(8.09, 11.41)	<0.001	9.71	(8.05, 11.37)	<0.001
Helicopter transport (yes)	1.68	(0.51, 2.86)	0.005	3.51	(2.40, 4.61)	<0.001	3.54	(2.44, 4.65)	<0.001

Patients with a NACA score of 4–6 and acute myocardial infarction, stroke, head injury, or penetrating torso injury (N = 1605) were included. ^a^Year in study period refers to year 1–5 of the period from 2009 to 2013. ^b^Variables chosen for final model included multivariate regression analyses of variables that differed significantly, in addition to age and gender. The fully adjusted model is an intermediate calculation step to select factors for the final model. Estimates were adjusted for HEMS base, and the individual physician was used as a random effect. B = unstandardized coefficient in the regression model (minutes per unit of predictor). Positive values are associated with increased on-scene time. Fully adjusted model is an intermediate calculation step to select factors for final model. Missing values: 6 for age and 2 for daylight. OST was missing in 37 (2.3%) of the 1642 identified missions in the four included subgroups

No significant differences in OST were found for season of the year, month, day of the week, time of day, Revised Trauma Score, or between the three HEMS bases. Missing values are presented in Additional file 8.

## Discussion

We found a short OST in preselected conditions compared to other studies [4, 21–23]. For the various patient subgroups, the strength of association between factors and OST varied. However, none of the directions of effects changed between the subgroups with an anticipated short OST. Reducing the prehospital time is important in many severe medical emergencies and trauma, but the ideal OST cannot be stated for all conditions, such as when patient access is a challenge due to entrapment or in water or mountain rescue [24, 25].

A short OST will not reduce morbidity or mortality for any given patient. Even in our four subgroups of severe conditions with definitive care only available in hospital, reducing the OST may not affect survival for most patients. A recent Norwegian study reported that HEMS was able to restore deranged physiology, even when prolonging on-scene time, in 240 emergency medical and trauma patients [26]. Newgard et al. found no association between OST and mortality in trauma patients with abnormal physiology [27]. Five years later, the same group reported that OST did not affect outcome in two cohorts including hemorrhagic shock and traumatic brain injuries. However, analysis of patients suffering hemorrhagic shock, showed an association between longer out-of-hospital time and mortality in subgroups of patients suffering blunt trauma or requiring in-hospital critical care [24]. Gonzales et al. reported correlation between prolonged prehospital time and increased patient mortality in rural vehicular trauma [8]. A review in 2015 presented inconsistent results of correlations between prehospital time intervals and different outcomes for trauma patients, including some studies reporting OST to be correlated with outcome in specific settings and conditions [2]. It is difficult to identify which of the severely ill or injured patients that will benefit from a short OST. Hence, the appropriate approach may be to strive for a short OST in all critically ill patients where definitive care is only available in the hospital. The valuable scene time should only be spent for necessary assessments and interventions to avoid immediate threats to life and prepare for safe transport.

Increasing severity (i.e., higher NACA score) prolonged the OST as anticipated, consistent with a study on paramedic-staffed ambulances responding to trauma only [28]. The same association was not found for reduced GCS values in the final analysis in the linear mixed effect model. However, in the univariate analysis, a lower GCS value had a strong effect. Thus, some of the other factors better explained the variation in OST.

If a patient was transported by helicopter, the OST also increased. Interventions and preparations are often required before flight because of limited resources and space available in the helicopter, increasing OST [29, 30]. In ground ambulance transports with the HEMS physician attending, many interventions can be performed during transport instead of on-scene. However, this decrease in OST must be balanced against increased transport time.

We anticipated altered OST when advanced interventions were performed on-scene. Half of the patients with a NACA score of 4–6 received advanced interventions on-scene or during transport. Intubation by the HEMS had a large impact on OST. Yet, the median OST was rather short even when the patient was intubated, highlighting our focus on a short OST in severe conditions with need for in-hospital interventions. The unexpected low proportion of intubations in head injuries most probably reduced the median OST in this subgroup. A German study reported a mean OST of nearly 40 min, and a large proportion of their patients (65.7%) were intubated on-scene [30]. The large difference from our study probably reflects different on-scene priorities rather than differences in the patients’ conditions. Even though severe conditions or deranged physiology make the HEMS strive to immediately start transportation from the scene, rapid initiation of transport without assessing the airway, breathing, and circulation may decrease survival. The necessity of performing a given intervention during a particular mission cannot be determined in our retrospective design, but identifying the interventions that justify increased time on-scene could be an interesting aim for prospective studies. In some cases, the best choice may be to avoid interventions due to the short transport time to the hospital. We think that an experienced HEMS physician trained to make these crucial decisions is a major advantage.

Previous studies assessing whether HEMS interventions alter the OST are heterogeneous and report contradictory results [2, 9–13, 22, 24, 26, 31, 32]. Intravenous access is often established prior to HEMS arrival, but we found a significantly increased OST in missions with patients in need of intravenous analgesics. The time needed for the administration of drugs, as well as the evaluation of its effect, may explain the increased OST. In contrast, the decreased OST when using intravenous analgesics in cardiac arrest probably indicates that patients were successfully resuscitated in a shorter time and were responsive during the mission.

When advanced treatment is necessary prior to helicopter flights (i.e., intubation, thoracostomy, etc.), the time spent on-scene will unavoidably increase [13]. This is particularly important if paramedics on-scene cannot perform the intervention prior to HEMS arrival; thus, OST is also influenced by local treatment protocols. This may explain why we found no increased OST for patients with acute myocardial infarction when choosing helicopter flight. In most such cases, treatment protocols (e.g., drugs, ECG) were already followed by paramedics or general practitioners before HEMS arrival. This was confirmed by the OST in acute myocardial infarction being reduced if treatment before HEMS arrival was reported.

The OST increased by 2 s for each year increase in patient age, assuming that the variable was linear. No information was available on patients’ morbidity prior to the incident, but we think the patient’s current condition is more important and this small increase in OST reflects comorbidity rather than the age itself. The decrease in OST (2 min from the first to the last study year) can be explained by an increased focus on reducing OST in acute myocardial infarction and stroke. The factor “study year” differed significantly only in these two conditions, but these conditions represented three-fourths of the patients in the subgroups with an anticipated short OST.

The assessment and triage of critically ill patients by a qualified emergency physician, including transport to the appropriate level of care, is an advantage of the HEMS in our opinion. Our chosen method did not reveal an influence of physician experience on the OST. This effect is sparsely described in the literature. A German study reported increased prehospital times with junior physicians compared to senior physicians [33]. The advantage of a physician attending on-scene is debatable, but it does not seem to prolong the OST [6, 7, 34–38]. Physician-staffed HEMS services can speed-up the decision to depart from the scene, but may also increase the OST due to more advanced interventions being performed. The diagnostic competence and clinical decision-making are important assets of our HEMS. On-scene decisions made by the prehospital team also demand both technical and non-technical skills [39, 40]. If rapid transport is prioritized, a trained HEMS physician accompanying the patient to the hospital can provide more targeted interventions depending on the patient’s condition and acute needs. The impact of physicians’ skills and experience on the OST deserves further investigation.

Contrary to what we assumed, no difference was found in the OST of day versus night missions. We also did not find any difference in the OST in regards to the time of day. This may be due to the treatment protocols and operating procedures used by our services, the crews being accustomed to challenging weather and darkness, and that an ambulance was on-scene to assist the HEMS in most cases and able to provide artificial light. A German study reported darkness as a significant factor for increased OST.

Reported factors affecting the OST in all missions by a service are of little interest due to the large variation between different patient groups. The HEMSs in the US, Canada, Australia, and Europe differ greatly in crew composition, service hours, mission types, patient conditions, and on-scene strategy; therefore, different OSTs are reported (8 to 40 min) [9, 21, 28, 30, 41, 42]. Comparisons to such heterogeneous studies are challenging. In our data, the median OST was 4-times greater in cardiac arrest cases than patients with penetrating torso injuries. Therefore, if missions with cardiac arrest are included in reports on the OST for a service, important factors affecting OST can be neglected.

A strength of our study is the definition of OST used, which does not include the time used for shut-down, loading, and start-up of the helicopter. This definition is different from most other studies, which define OST as the time from the helicopter landing to take-off. However, this may have reduced our OST compared to other studies. Another strength of this study was the multicenter design involving three different HEMS bases with a similar national HEMS profile. The retrospective design provided a large number of missions, which allowed us to analyze the OST in specific and severe conditions and assess whether factors affecting OST varied between different patient conditions. Finally, we excluded conditions with NACA scores of 0–3 or 7 from most analyses, as increased OST among these patients is not likely to be associated with worse patient outcomes.

## Limitations

A retrospective design has weaknesses, such as misclassifications (e.g., failure to report patient entrapment in missions associated with increased OST). Missing data are another challenge. In our HEMS, no data were recorded automatically. In a large proportion of missions, GCS was not registered. It is not mandatory to register GCS on every patient in our HEMS, and it is often not registered when encountering awake and alert patients. We replaced missing GCS data with normal values to avoid losing half of the cases in the regression analysis. This may have increased the likelihood of a type 2 error, but not a type 1 error. Our database does not differentiate between interventions on-scene or during transport; thus, some of the interventions performed may not have influenced the OST. Many of the excluded variables may have had an impact on OST but were unavailable for analysis; for example, in a road traffic accident with several patients, the actual number of patients assessed on-scene probably affects the OST. For patients who died on-scene or during transport (NACA score = 7), OST was recorded in only 133 missions (12%) and probably reflects the missions in which transport was initiated but the patient died before arriving at the hospital. No major HEMS changes were introduced during the study period.

Conclusive data on patient outcomes after HEMS treatment are needed. Given the costs involved, tools should be developed to better identify patients who will benefit the most from this service. As geographical challenges and regional organization (e.g., operational pattern, patient referral system, and resource availability) may differ from other HEMSs, generalizations from our study must be made with caution.

## Conclusions

We have demonstrated a rather short OST in our service compared to other published studies. The time spent on-scene and its influencing factors were dependent on the patient’s condition and shortest in penetrating torso injuries. The most important factors associated with an increased OST were the severity of the patient’s condition, the recorded use of endotracheal intubation or intravenous analgesics, helicopter transport, and trauma missions. Treatment prior to HEMS arrival reduced OST in patients suffering from acute myocardial infarction or stroke. Our results provide a basis for efforts to improve decision making and reduce OST for selected patient groups.

## Additional files

Additional file 1: Patient characteristics and on-scene time (OST) in primary emergency missions with entrapped patient (*N* = 41). (XLS 27 kb)

Additional file 2: The NACA scale; a severity scoring used by the Norwegian Air Ambulance Service. (XLS 28 kb)

Additional file 3: Factors affecting on-scene time (linear mixed effect model) in acute myocardial infarction. (XLS 33 kb)

Additional file 4: Factors affecting on-scene time (linear mixed effect model) in stroke. (XLS 33 kb)

Additional file 5: Factors affecting on-scene time (linear mixed effect model) in head injuries. (XLS 33 kb)

Additional file 6: Factors affecting on-scene time (simple linear model) in penetrating torso injuries. (XLS 33 kb)

Additional file 7: Factors affecting on-scene time (linear mixed effect model) in cardiac arrest. (XLS 33 kb)

Additional file 8: Missing registrations of variables in the 9757 missions in Table 1. (XLS 31 kb)

## Abbreviations

ECG: Electrocardiography
EMCC: Emergency Medical Communication Centre
GCS: Glasgow Coma Scale
HEMS: Helicopter emergency medical service
IQR: Interquartile Range
NACA: National Advisory Committee for Aeronautics
OST: On-scene time
SAR: Search- and Rescue
